# Case report: Chronic inflammatory demyelinating polyneuropathy superimposed on Charcot–Marie-tooth type 1A disease after SARS-CoV-2 vaccination and COVID-19 infection

**DOI:** 10.3389/fneur.2024.1358881

**Published:** 2024-04-08

**Authors:** Da Li, Hu Yu, Min Zhou, Weinv Fan, Qiongfeng Guan, Li Li

**Affiliations:** Department of Neurology, Ningbo No 2 Hospital, Ningbo, Zhejiang, China

**Keywords:** Charcot–Marie–tooth disease, chronic inflammatory demyelinating polyneuropathy, peripheral myelin protein 22, SARS-CoV-2, case report

## Abstract

**Background:**

There is growing evidence that severe acute respiratory syndrome coronavirus-2 (SARS-CoV-2) or COVID-19 infection is associated with the development of immune mediated neuropathies like chronic inflammatory demyelinating polyneuropathy (CIDP), but the impact of SARS-CoV-2 vaccination and COVID-19 infection on genetic disorders such as Charcot–MarieTooth (CMT) remains unclear.

**Case presentation:**

A 42-year-old male with occulted CMT neuropathy type lA (CMT1A) who developed limb numbness and weakness after the second SARS-CoV-2-vaccination was confirmed by identifying characteristic repeats in the p11.2 region of chromosome 17. Due to the progressive deterioration of muscle strength over 8 weeks, limb atrophy, moderately elevated protein counts in the cerebrospinal fluid, and significant improvement with intravenous human immunoglobulin, which were characteristic of acquired inflammatory neuropathies, he was eventually diagnosed with CIDP superimposed on CMT1A. However, after a three-month plateau, the patient contracted COVID-19, which led to repeated and worsening symptoms of limb weakness and atrophy, thus was diagnosed with a recurrence of CIDP and treated with Intravenous immunoglobulin and methylprednisolone 500 mg/d for 5 consecutive days, followed by oral prednisone and mycophenolate mofetil tablets. On 2 month follow-up, he exhibited remarkable clinical improvement and could walk independently with rocking gait. After 1 year of follow-up, the patient’s condition was stable without further change.

**Conclusion:**

Our case indicates that CMT1A can deteriorate after SARS-CoV-2 vaccination. Thus, SARS-CoV-2 vaccination should be considered a potential predisposing factor for CMT1A worsening. The possible superposition of CMTIA and CIDP in the context of SARS-CoV-2 infection or immunity suggests that any clinical exacerbation in patients with CMT1A should be carefully evaluated to rule out treatable superposition inflammation. In addition, electrophysiological and imaging examination of the proximal nerves, such as the axillary nerve, is helpful for the diagnosis of CIDP.

## Background

The spectrum of reported neurological sequelae associated with severe acute respiratory syndrome coronavirus-2 (SARS-CoV-2) vaccination or COVID-19 infection is continuously expanding, facial nerve palsy (1), Guillain Barré syndrome, chronic inflammatory demyelinating polyneuropathy (CIDP) and myasthenic disorders being among them (2–4), but the impact of vaccination and COVID-19 infection on genetic disorders such as Charcot–Marie Tooth (CMT) are unclear at present.

CMT is a subgroup of inherited neuropathies in which peripheral nerve involvement is the sole or predominant feature (5). Some researchers have speculated that this genetic defect may make patients more susceptible to acquiring a secondary inflammatory neuropathy (6, 7), resulting in CMT superimposed with CIDP. After infection or inflammation, CIDP may be superimposed on some forms of CMT (7–9), which can be effectively relieved with treatment.

Herein, we first report a patient with occulted CMT1A due to a mutation in the peripheral myelin protein 22 (PMP22) gene who developed severe recurrent neuropathy after a previous SARS-CoV-2 vaccination and subsequent COVID-19 infection. He possessed the clinical and neurophysiological features of CIDP and had a marked response to immunotherapy.

## Case presentation

A 42-year-old previously healthy man received his second dose of an inactivated SARS-CoV-2 vaccine (Beijing Institute of Sinovac Products Co., Ltd., Beijing, China) on July 11th, 2021. One week later, he developed paresthesia of the feet and hands, associated with distal weakness in the wrists and lower limbs below the knee joint, which evolved over a period of 6 months. He reported impaired manual dexterity and gait unsteadiness with increasing difficulties being faced in his work. He had a good general health status with a weight of 80.0 kg, height of 1.75 m, BMI of 26.12 Kg/m^2^, no other complaints, and his family history was unremarkable. Upon admission to our hospital, neurological examination showed normal cranial nerve and cognitive function. His Medical Research Council (MRC) score for strength was 4+/5 on bilateral extension, abduction of fingers, hip flexion, and ankle and toe dorsiflexion. Proprioception and light touch sensation were intact. Tendon reflexes of the lower limbs and upper limbs were not elicited and were decreased, respectively. Mild interosseous muscle atrophy and bilateral pes cavus without evident peroneal atrophy were noted.

A subsequent electrophysiological study showed that the compound muscle action potentials of the median, ulnar, tibial, and peroneal nerves were all absent. The distal latencies of the axillary nerve were increased (9.1 ms on the right, 9.5 ms on the left) with low amplitude compound muscle action potentials (4.9 mV on the right, 4.7 mV on the left). Sensory action potentials were absent in both the median and sural nerves. F-waves were unobtainable (Table 1). On electromyography (EMG), fibrillation potentials and positive sharp waves were present in the left extensor digitorum communis and gastrocnemius, right interosseous muscle, and tibialis anterior. Neurophysiologic evaluation showed severe demyelination with axonal sensorimotor polyneuropathy, symmetrical upper and lower extremities, and more distal involvement. Nerve high resolution ultrasound showed an increase of the cross-sectional area at all nerves in the extremities. Brachial 3.0 T magnetic resonance imaging (MRI) revealed mild swelling of the bilateral brachial plexuses without marked gadolinium-DTPA (diethylenetriamine penta-acetic acid) enhancement of the nerve ganglion (Supplementary material 1).

**Table 1:** NCS results in the patient.

Motor conduction																			Sensory conduction	
		Median N					Ulnar N					Peroneal N					Axillary N		Sural N	
		Wrist-APB		Elbow-wrist			Wrist-ADM		Elbow-wrist			Ankle-EDB		Knee-ankle			Erb’s-deltoid			
Testing Time		DML (ms)	cMAP (mV)	cMAP (mV)	MNCV (m/s)	F-wave (ms)	DML (ms)	cMAP (mV)	cMAP (mV)	MNCV (m/s)	F-wave (ms)	DML (ms)	cMAP (mV)	cMAP (mV)	MNCV (m/s)	F-wave (ms)	DML (ms)	cMAP (mV)	SAP (μV)	SNCV (m/s)
2021–12	R		0	0		n.e.		0	0		n.e.		0	0		n.e.	9.1	4.9	0	
	L		0	0		n.e.		0	0		n.e.		0	0		n.e.	9.5	4.7	0	
2022–09*	R	13.6	0.035	0.04	12.4	n.e.	8.71	0.27	0.13	17.1	14.7		0	0.78	14.3	n.e.	-	-	0	
	L		0	0		n.e.		0	0		n.e.		0	0		n.e.	-	-	0	
2023–02	R		0	0		n.e.		0	0		n.e.		0	0		n.e.	7.6	0.857	0	
	L		0	0		n.e.		0	0		n.e.		0	0		n.e.	9.8	1.146	0	

ADM, abductor digiti minimi; APB, abductor pollicis brevis; cMAP, compound muscle action potential; DML, distal motor latency; EDB, extensor digitorium brevis; MNCV, motor nerve conduction velocity; n.e., not evoked; SAP, Sensory action potential; *The second EMG data was re-examined by an experienced electrophysiologist at another top hospital.

Cerebrospinal fluid (CSF) analysis exhibited albuminocytological dissociation (protein 0.961 g/L, 1 cell) with no oligoclonal banding on immunofixation. The CSF paraneoplastic panel, flow cytometry, and cytopathology were within normal limits. Other investigations to determine the causes were conducted. Cardiac ultrasound, head MRI, cervical MRI, electroencephalogram, and lower extremity vascular results were unremarkable. Serum determinations of perinuclear staining antineutrophil cytoplasmic antibody (P-ANCA), antinuclear antibodies, myeloperoxidase antibodies, cytoplasmic-ANCA and proteinase 3, rheumatoid factor, anti-O, alpha-fetoprotein, carcinoembryonic antigen, myelin associated glycoprotein, serum and urine protein immunofixation electrophoresis, serum and urine light chain, thyroid function tests, vitamin B12, and antibodies against hepatitis viruses were normal or negative; anti-ganglioside, anti-neuronal nuclear and plasma membrane antibodies, and titrations for syphilis, and HIV antibodies were negative in the blood specimens. His fasting blood-glucose (7.31 mmol/L) and glycosylated hemoglobin (9.9%) were elevated, suggesting a new onset of diabetes. Due to severe neuropathy, he was immediately scheduled for extensive genetic testing. Multiplex ligation-dependent probe amplification analysis for investigating PMP22 gene deletion/duplication was performed, finally, duplication of the p11.2 region of chromosome 17, the hallmark of CMT1A, was found.

Over the next 9 months, treatment with B vitamins and blood glucose control was unsuccessful, and his condition worsened with progressive numbness, weakness of the limbs and muscle atrophy of the hand. He had bilateral pes cavus. Motor examination revealed mild distal upper (MRC grade 4/5) and distal severe lower limb (2/5) symmetrical weakness. The CSF was acellular with a total protein concentration of 1.323 g/L. Serum and CSF testing for antiganglioside antibodies and antibodies directed against myelin or proteins localized to the Ranvier node were negative. The report by a top electrophysiologist at another hospital in Shanghai, China, showed multiple peripheral nerve damage involving demyelination of motor and sensory nerves with axonal damage (Table 1). Overall, findings were compatible with inflammatory peripheral nervous system disorder and the presentation was thought to be a post-vaccination reaction. After diagnosing CIDP superimposed on CMT1A, intravenous human immunoglobulin (IVIg) was administered at a standard dosage (0.4 g/kg over five consecutive days), followed by oral mattemycophenol ester 0.75 g bid and prednisone 50 mg qd. After a month his clinical condition improved [corresponding to two points on the score in the Inflammatory Neuropathy Cause and Treatment (INCAT) disability scale (10)]. He regained hand function, walked freely. He stopped taking oral medication and remained clinically stable during the following 3 months.

**Figure 1:**
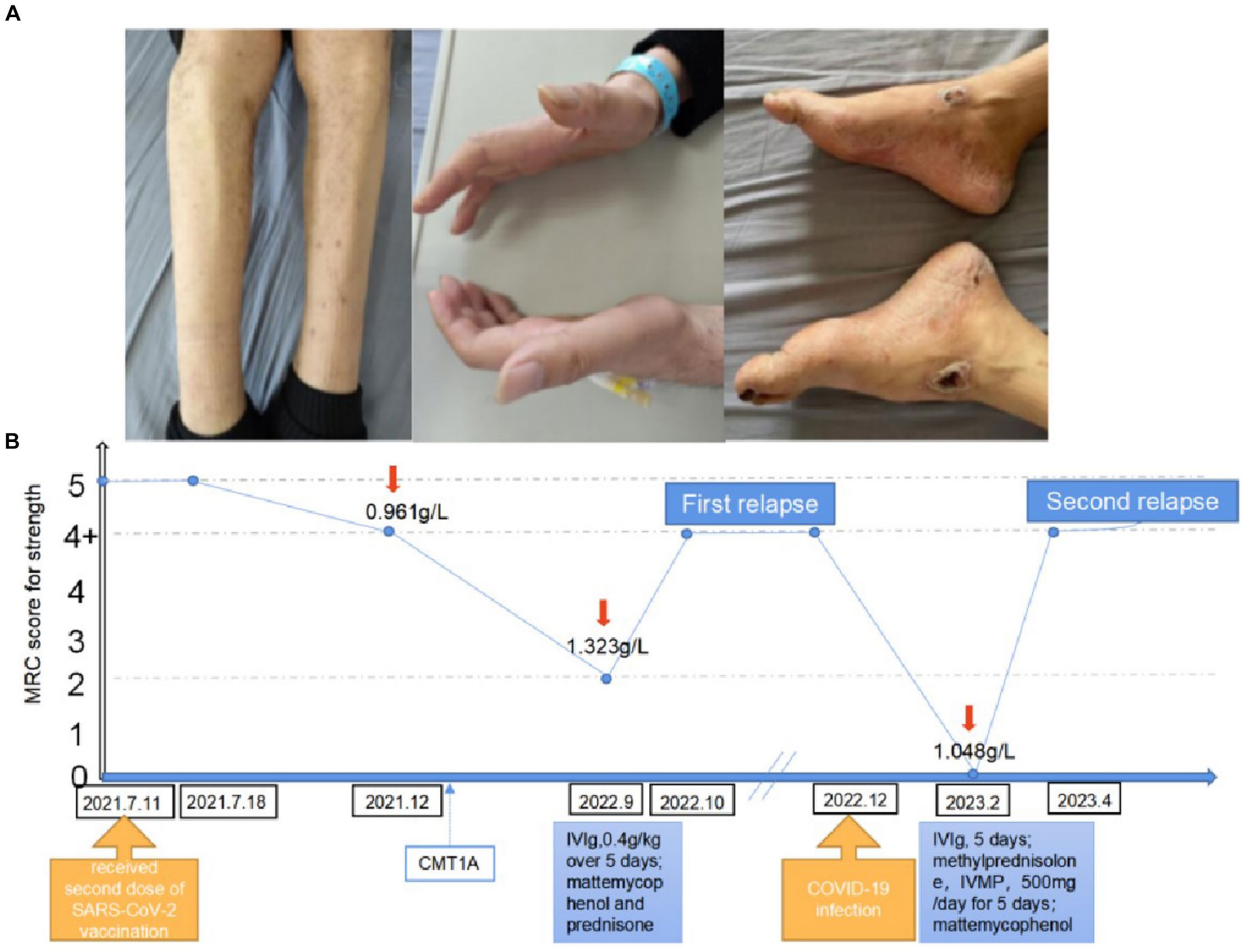
**(A)** Patient phenotypic characteristics with pes cavus, hammer toes and hand muscles atrophy. **(B)** Timeline with clinical course, the red arrow indicates the CSF protein counts. IVIg, intravenous immunoglobulin; CMTIA, Charcot-MarieT00th neuropathy type IA; SARS-COV-2, severe acute respiratory syndrome coronavirus-2; IVMP, intravenous methylprednisolone.

Unfortunately, after a 3-month plateau, the patient was infected with COVID-19 (confirmed by nasopharyngeal swab PCR). About 1 week thereafter, the patient had a relapse, with severe, rapidly progressive quadriparesis and hand and feet numbness lasting over 8 weeks, that lead to loss of ambulation. His muscle strength was 4/5 in the proximal lower and upper limbs, and was 0/5 in the distal lower limbs. The patient had significant weight loss (10 kg). Neurologic examination revealed bilateral pes cavus, marked peroneal and interosseous muscle atrophy (Figure 1A), bilateral weakness of ankle dorsiflexors with foot drop, superficial sensory loss in a stocking distribution, and abolished vibration sense and joint position sense at the distal extremities of both lower limbs. CSF analysis disclosed moderately raised protein counts (1.048 g/L) with no pleocytosis. Neuroelectrophysiology and brachial plexus magnetic resonance data were similar to those obtained 14 months prior.

He, once again, had a relatively rapid and progressive clinical improvement with IVIg (27.5 g per day for 5 days) and intravenous methylprednisolone (500 mg/day for 5 days), which were started 9–10 weeks into this new relapse. The intravenous medication was gradually replaced by oral drugs (corticoids). He was discharged from hospital and oral immunosuppressants were added (mycophenolate mofetil tablets). There was a gradual restoration of limb movement and locomotion (with support). The INCAT disability scale score was reduced by 1 point. The timeline with clinical course is shown in Figure 1B.

One year after discharge (2024.2), the patient’s distal limb numbness and weakness did not worsen, and he did not take any drugs. He usually used bilateral support to walk outdoors, fine movements of both hands were not flexible, and daily life is partially self-reliant.

## Discussion and conclusion

Prednisone responsiveness has been reported in a few cases of hereditary neuropathies, of which an inflammatory component of demyelination seemed to be superimposed (11–13). Furthermore, the presence of perivascular lymphocytic infiltrates and macrophages in nerve biopsies of patients with CMT proves the occurrence of a superimposed inflammatory process (11). Acute inflammatory demyelinating polyradiculoneuropathy, CIDP, and inflammatory ataxic neuropathy have been described in association with CMT1A (14). In our case, this patient fulfilled the following definite criteria for CIDP based on EFNS/PNS and China guidelines (15, 16) except for the co-occurrence of a hereditary neuropathy (17): 1. typical clinical course progression of more than 8 weeks; 2. electrodiagnostic evidence of demyelinating lesions of 2 nerves; 3. three of the four supportive criteria, including effective immunotherapy, elevated CSF protein, and increased cross-sectional areas of the median, ulnar and brachial plexus as confirmed by peripheral nerve ultrasound and MRI. Since he tested positive for the PMP22 duplication on chromosome 17, we made the diagnosis of CIDP superimposed on CMT1A. As we found no other etiologies and due to close temporal relationship, we suspect the SARS-CoV-2 vaccination as a potential predisposing factor for CMT1A deterioration. Simultaneously, in the context of this genetic disease, COVID-19 triggered the superimposition and recurrence of CIDP. To the best of our knowledge, this is the first report of a CMT1A overlapping CIDP occurring in the context of the dual action of SARS-CoV-2 vaccination and COVID-19 infection, causing the deterioration of neurological disability in a case of previously mild demyelinating hereditary neuropathy.

In CMT, it appears that coexisting inflammatory neuropathy is not genotype-specific. Most reports involve PMP22, MPZ (18, 19), and the X-linked CMT gene (20, 21), which are key elements in the formation of compact myelin (22), suggesting that the development of an inflammatory process in CMT disease may relate to a disturbance in the normal function of the protein encoded by the affected gene (7, 14, 23). Duplication of the PMP22 gene is the most common cause of CMT1A neuropathy. For patients with chromosome 17p11.2 ± 12 duplication, it has been suggested that there may be genes within the duplicated segment which are capable of modifying the immune response (23).

In our case, the electrophysiology and muscle strength changes in the early disease stage were highly inconsistent, which is a rare clinical phenomenon, but it also exists in CMT1 with other gene mutation (24), and the specific mechanism is unknown. Recently, copy number variants have been found to be responsible for the phenotypes of numerous inherited disorders (25, 26). Several researchers have reported a triplication of the 17p12 locus in some patients with a severe CMT1A phenotype (27). PMP22 gene mutations are the most likely pathogenic mechanism underlying the severity of CMT1A (28, 29). Preclinical model suggested that the phenotypical severity of P0+/− mice was mitigated by an immunodeficient genetic background (30). These above evidences suggests that the clinical phenotypic severity of CMT may be related to the type of gene mutation, copy number variants and immune composition.

CIDP developing in the post-vaccination period is distinctly unusual, accounting for about 1.5% of patients (31), whereas CMT overlapping CIDP associated with vaccine and viral infection is even rarer and has been observed in only one patient due to recent HIV infection (9). It has been proposed that a postvaccination neurological syndrome could result from the generation of host antibodies that cross-react with proteins present in peripheral myelin (32). Because there is evidence of a cross-reactivity between the SARS-CoV-2 spike protein and peripheral nerve glycolipids (32, 33), these antibodies may be generated in direct response to the SARS-CoV-2 spike protein. The role of vaccination in formulating host immunity against certain pathogens may conversely stimulate the progression of preexisting subclinical autoimmunity to an overt disease.

With the advances in molecular analysis and neuroimaging techniques (24, 34) in the last decade, most patients with a genetic basis for their neuropathy can now be given a firm and specific diagnosis. Similarly, strict criteria now exist for the diagnosis of CIDP, emphasizing neurophysiological measurements. But in the condition of superimposition, many electrophysiological features of acquired demyelination are potentially being masked by the chronic changes of hereditary diseases, in which case it is necessary to combine neuroelectrophysiological detection, neuroimaging, CSF detection, and nerve biopsy for identification (15). In this case, the diagnosis of CIDP is based on both clinical criteria (relapse and remission following intravenous immunoglobulins) and neurographic criteria. Electrophysiological examination of proximal nerves such as the axillary nerve is helpful for the diagnosis of CIDP.

In a recent literature review, it was suggested that the CSF protein content, plexus MRI, and clinical progression features can be used to distinguish between hereditary and inflammatory neuropathy (14). An immune process was suspected due to the presence of elevated CSF proteins, stepwise recurrent exacerbation, and a partial and limited response to steroids. The recognition of an inflammatory component creates therapeutic opportunities for some CMT patients. Albeit, there is no cure for inherited demyelinating neuropathies. We treated our patient according to the available recommendation with IVIg administration at the earliest convenience, which induced an evident clinical amelioration (35). The optimal management and long-term outcomes of these patients remain to be determined (9).

In conclusion, the association of CMT1A and CIDP comorbidity with COVID-19 vaccine and infection has not been reported previously and might be considered fortuitous, but it is more likely that a genetic predisposition favors the development of acquired inflammatory demyelinating processes. Our report expands on the possible outcomes in patients after COVID-19, providing evidence for the prevention and treatment of COVID-19 in patients with genetic disorders. In the post-pandemic era, which can turn into flu-like normalization, neurologists need to follow-up and carefully assess the evolution of patients with various genetic disorders after infection. The identification of inflammatory components may improve some clinical symptoms in patients with genetic diseases.

## Data availability statement

The raw data supporting the conclusions of this article will be made available by the authors, without undue reservation.

## Ethics statement

The studies involving humans were approved by Ningbo Second Hospital Ethics Committee. The studies were conducted in accordance with the local legislation and institutional requirements. Written informed consent for participation was not required from the participants or the participants’ legal guardians/next of kin in accordance with the national legislation and institutional requirements. Written informed consent was obtained from the individual(s) for the publication of any potentially identifiable images or data included in this article. Written informed consent was obtained from the participant/patient(s) for the publication of this case report.

## Author contributions

DL: Formal analysis, Investigation, Methodology, Writing – original draft. HY: Data curation, Investigation, Supervision, Writing – review & editing. MZ: Writing – review & editing. WF: Funding acquisition, Project administration, Supervision, Writing – review & editing. QG: Funding acquisition, Resources, Validation, Writing – review & editing. LL: Investigation, Supervision, Validation, Writing – review & editing.

## References

[1] Panholzer J Kellermair L Eggers C. Hypoglossal nerve palsy after SARS-CoV-2 vaccination - report of two cases. BMC Neurol. (2022) 22:416. doi: 10.1186/s12883-022-02929-2, PMID: 36352369 PMC9643981

[2] Patone M Handunnetthi L Saatci D Pan J Katikireddi SV Razvi S. Neurological complications after first dose of COVID-19 vaccines and SARS-CoV-2 infection. Nat Med. (2021) 27:2144–53. doi: 10.1038/s41591-021-01556-7, PMID: 34697502 PMC8629105

[3] Iacono S Di Stefano V Alonge P. Adherence and Reactogenicity to vaccines against SARS-COV-2 in 285 patients with neuropathy: a multicentric study. Brain Sci. (2022) 12:1396. doi: 10.3390/brainsci12101396, PMID: 36291329 PMC9599423

[4] Ginanneschi F Vinciguerra C Volpi N Piscosquito G Barone P Rossi A. Chronic inflammatory demyelinating polyneuropathy after SARS-CoV2 vaccination: update of the literature and patient characterization. Immunol Res. (2023) 71:833–8. doi: 10.1007/s12026-023-09406-z, PMID: 37395901

[5] Rossor AM Evans MR Reilly MM. A practical approach to the genetic neuropathies. Pract Neurol. (2015) 15:187–98. doi: 10.1136/practneurol-2015-001095, PMID: 25898997

[6] Cottenie E Menezes MP Rossor AM Morrow JM Yousry TA Dick DJ. Rapidly progressive asymmetrical weakness in Charcot-Marie-tooth disease type 4J resembles chronic inflammatory demyelinating polyneuropathy. Neuromuscul Disord. (2013) 23:399–403. doi: 10.1016/j.nmd.2013.01.010, PMID: 23489662

[7] Ginsberg L Malik O Kenton AR Sharp D Muddle JR Davis MB. Coexistent hereditary and inflammatory neuropathy. Brain. (2004) 127:193–202. doi: 10.1093/brain/awh017, PMID: 14607795

[8] Campagnolo M Taioli F Cacciavillani M Ruiz M Luigetti M Salvalaggio A. Sporadic hereditary neuropathies misdiagnosed as chronic inflammatory demyelinating polyradiculoneuropathy: pitfalls and red flags. J Peripher Nerv Syst. (2020) 25:19–26. doi: 10.1111/jns.12362, PMID: 31919945

[9] Rajabally Y Vital A Ferrer X Vital C Julien J Latour P. Chronic inflammatory demyelinating polyneuropathy caused by HIV infection in a patient with asymptomatic CMT 1A. J Peripher Nerv Syst. (2000) 5:158–62. doi: 10.1046/j.1529-8027.2000.00014.x, PMID: 11442172

[10] Breiner A Barnett C Bril V. INCAT disability score: a critical analysis of its measurement properties. Muscle Nerve. (2014) 50:164–9. doi: 10.1002/mus.24207, PMID: 24723454

[11] Bird SJ Sladky JT. Corticosteroid-responsive dominantly inherited neuropathy in childhood. Neurology. (1991) 41:437–9. doi: 10.1212/wnl.41.3.437, PMID: 1848688

[12] Malandrini A Villanova M Dotti MT Federico A. Acute inflammatory neuropathy in Charcot-Marie-tooth disease. Neurology. (1999) 52:859–61. doi: 10.1212/wnl.52.4.859 10078742

[13] Gazulla J Almarcegui C Berciano J. Reversible inflammatory neuropathy superimposed on Charcot-Marie-tooth type 1A disease. Neurol Sci. (2018) 39:793–4. doi: 10.1007/s10072-017-3195-z, PMID: 29164357

[14] Rajabally YA Adams D Latour P Attarian S. Hereditary and inflammatory neuropathies: a review of reported associations, mimics and misdiagnoses. J Neurol Neurosurg Psychiatry. (2016) 87:1051–60. doi: 10.1136/jnnp-2015-310835, PMID: 27010614

[15] Van den Bergh P van Doorn PA Hadden R. European academy of neurology/peripheral nerve society guideline on diagnosis and treatment of chronic inflammatory demyelinating polyradiculoneuropathy: report of a joint task force-second revision. Eur J Neurol. (2021) 28:3556–83. doi: 10.1111/ene.14959, PMID: 34327760

[16] Chinese Society of Neurology, Peripheral neuropathy collaboration Group of Chinese Society of neurology, Chinese Society of Electromyography and Clinical Neuroelectrophysiology. The guideline of diagnosis and treatment of chronic inflammatory demyelinating polyradiculoneuropathy. Chin J Neurol. (2023) 56:125–32. doi: 10.3760/cma.j.cn113694-20220627-00507

[17] van den Bergh P Hadden RD Bouche P Cornblath DR Hahn A Illa I. European Federation of Neurological Societies/peripheral nerve society guideline on management of chronic inflammatory demyelinating polyradiculoneuropathy: report of a joint task force of the European Federation of Neurological Societies and the peripheral nerve society - first revision. Eur J Neurol. (2010) 17:356–63. doi: 10.1111/j.1468-1331.2009.02930.x, PMID: 20456730

[18] Donaghy M Sisodiya SM Kennett R McDonald B Haites N Bell C. Steroid responsive polyneuropathy in a family with a novel myelin protein zero mutation. J Neurol Neurosurg Psychiatry. (2000) 69:799–805. doi: 10.1136/jnnp.69.6.799, PMID: 11080236 PMC1737181

[19] Watanabe M Yamamoto N Ohkoshi N Nagata H Kohno Y Hayashi A. Corticosteroid-responsive asymmetric neuropathy with a myelin protein zero gen mutation. Neurology. (2002) 59:767–9. doi: 10.1212/wnl.59.5.767, PMID: 12221176

[20] D'Urso D Brophy PJ Staugaitis SM Stewart Gillespie C Frey AB Stempak JG. Protein zero of peripheral nerve myelin: biosynthesis, membrane insertion, and evidence for homotypic interaction. Neuron. (1990) 4:449–60. doi: 10.1016/0896-6273(90)90057-m, PMID: 1690568

[21] Filbin MT Walsh FS Trapp BD Pizzey JA Tennekoon GI. Role of myelin P0 protein as a homophilic adhesion molecule. Nature. (1990) 344:871–2. doi: 10.1038/344871a0 1691824

[22] Lemke G. Unwrapping the genes of myelin. Neuron. (1988) 1:535–43. doi: 10.1016/0896-6273(88)90103-1, PMID: 2483101

[23] Gabriel CM Gregson NA Wood NW Hughes RA. Immunological study of hereditary motor and sensory neuropathy type 1a (HMSN1a). J Neurol Neurosurg Psychiatry. (2002) 72:230–5. doi: 10.1136/jnnp.72.2.230, PMID: 11796774 PMC1737757

[24] Cardellini D Zanette G Taioli F Bertolasi L Ferrari S Cavallaro T. CIDP, CMT1B, or CMT1B plus CIDP? Neurol Sci. (2021) 42:1127–30. doi: 10.1007/s10072-020-04789-5, PMID: 33070202

[25] Salpietro V Manole A Efthymiou S Houlden H. A review of copy number variants in inherited neuropathies. Curr Genomics. (2018) 19:412–9. doi: 10.2174/1389202919666180330153316, PMID: 30258273 PMC6128387

[26] Cutrupi AN Brewer MH Nicholson GA Kennerson ML. Structural variations causing inherited peripheral neuropathies: a paradigm for understanding genomic organization, chromatin interactions, and gene dysregulation. Mol Genet Genomic Med. (2018) 6:422–33. doi: 10.1002/mgg3.390, PMID: 29573232 PMC6014456

[27] Pareyson D Testa D Morbin M Erbetta A Ciano C Lauria G. Does CMT1A homozygosity cause more severe disease with root hypertrophy and higher CSF proteins? Neurology. (2003) 60:1721–2. doi: 10.1212/01.wnl.0000059262.34846.8a, PMID: 12771282

[28] Gabriel JM Erne B Pareyson D Sghirlanzoni A Taroni F Steck AJ. Gene dosage effects in hereditary peripheral neuropathy. Expression of peripheral myelin protein 22 in Charcot-Marie-tooth disease type 1A and hereditary neuropathy with liability to pressure palsies nerve biopsies. Neurology. (1997) 49:1635–40. doi: 10.1212/wnl.49.6.1635 9409359

[29] Schenone A Nobbio L Mandich P Bellone E Abbruzzese M Aymar F. Underexpression of messenger RNA for peripheral myelin protein 22 in hereditary neuropathy with liability to pressure palsies. Neurology. (1997) 48:445–9. doi: 10.1212/wnl.48.2.445, PMID: 9040736

[30] Wang IC Kroner A Fischer S. Role of immune cells in animal models for inherited peripheral neuropathies. NeuroMolecular Med. (2006) 8:175–90. doi: 10.1385/nmm:8:1-2:175, PMID: 16775375

[31] Doneddu PE Bianchi E Cocito D Manganelli F Fazio R Filosto M. Risk factors for chronic inflammatory demyelinating polyradiculoneuropathy (CIDP): antecedent events, lifestyle and dietary habits. Data from the Italian CIDP database. Eur J Neurol. (2020) 27:136–43. doi: 10.1111/ene.14044, PMID: 31325350

[32] Allen CM Ramsamy S Tarr AW Tighe PJ Irving WL Tanasescu R. Guillain-Barre syndrome variant occurring after SARS-CoV-2 vaccination. Ann Neurol. (2021) 90:315–8. doi: 10.1002/ana.26144 34114269

[33] Fantini J Di Scala C Chahinian H. Structural and molecular modelling studies reveal a new mechanism of action of chloroquine and hydroxychloroquine against SARS-CoV-2 infection. Int J Antimicrob Agents. (2020) 55:105960. doi: 10.1016/j.ijantimicag.2020.105960, PMID: 32251731 PMC7128678

[34] Telleman JA Grimm A Goedee S Visser LH Zaidman CM. Nerve ultrasound in polyneuropathies. Muscle Nerve. (2018) 57:716–28. doi: 10.1002/mus.26029 29205398

[35] Leonhard SE Mandarakas MR Gondim F. Diagnosis and management of Guillain-Barre syndrome in ten steps. Nat Rev Neurol. (2019) 15:671–83. doi: 10.1038/s41582-019-0250-9, PMID: 31541214 PMC6821638

