# Corrigendum: Case report: Chronic inflammatory demyelinating polyneuropathy superimposed on Charcot–Marie-tooth type 1A disease after SARS-CoV-2 vaccination and COVID-19 infection

**DOI:** 10.3389/fneur.2024.1465995

**Published:** 2024-08-09

**Authors:** Da Li, Hu Yu, Min Zhou, Weinv Fan, Qiongfeng Guan, Li Li

**Affiliations:** Department of Neurology, Ningbo No 2 Hospital, Ningbo, Zhejiang, China

**Keywords:** Charcot–Marie–tooth disease, chronic inflammatory demyelinating polyneuropathy, peripheral myelin protein 22, SARS-CoV-2, case report

In the published article, there was an error in affiliation. Instead of “[Department of Neurology, Ningbo, Zhejiang, China]”, it should be “[Department of Neurology, Ningbo No 2 Hospital, Ningbo, Zhejiang, China]”.

The authors apologize for this error and state that this does not change the scientific conclusions of the article in any way. The original article has been updated.

