# Supplementary material for: Case report: Chronic inflammatory demyelinating polyneuropathy superimposed on Charcot–Marie-tooth type 1A disease after SARS-CoV-2 vaccination and COVID-19 infection
Source: Front Neurol. 2024 Apr 8;15:1358881. doi: 10.3389/fneur.2024.1358881 (PMC11033519; doi:10.3389/fneur.2024.1358881)
Supplement: Supplementary file 1 [file Image_1.pdf]

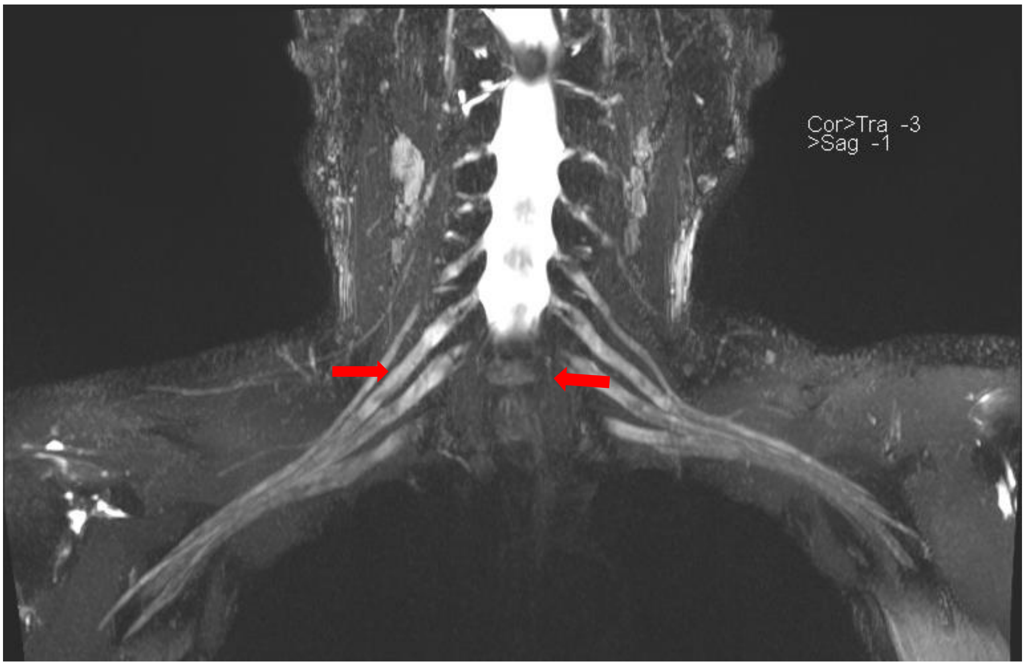

Fig.1. Coronal fat-suppressed T2-weighted images before treatment revealed mild swelling of the bilateral brachial plexuses (red arrow) without marked gadolinium-DTPA enhancement of the nerve ganglion.
